# Winter Rains Support Butterfly Diversity, but Summer Monsoon Rainfall Drives Post-Monsoon Butterfly Abundance in the Arid Southwest of the US

**DOI:** 10.3390/insects15010005

**Published:** 2023-12-21

**Authors:** Helen Ivy Rowe, Bradly Johnson, Jennifer Broatch, Terese Maxine Papag Cruz, Kathleen L. Prudic

**Affiliations:** 1 School of Earth and Sustainability, Northern Arizona University, Flagstaff, AZ 86011, USA; 2 Parsons Field Institute, McDowell Sonoran Conservancy, Scottsdale, AZ 85260, USA; 3 School of Mathematical and Natural Sciences, Arizona State University West, Phoenix, AZ 85069, USAjenn9@asu.edu (J.B.); 4 School of Natural Resources and the Environment, University of Arizona, Tucson, AZ 85721, USA; tmcruz@arizona.edu (T.M.P.C.); klprudic@arizona.edu (K.L.P.); 5 Arizona Institute for Resilience, University of Arizona, Tucson, AZ 85721, USA

**Keywords:** abundance, climate change, community science, precipitation, richness

## Abstract

**Simple Summary:**

Butterflies are in decline due to habitat conversion, climate change, and agricultural practices. Insects have a narrow range of habitable conditions, and extreme fluctuations in weather or climate can lead to population declines. Other studies have shown that butterfly populations are declining in western US; however, given the distinct climate of the southwest, with two distinct rainy seasons, we wanted to explore whether the general trends held true for Arizona. We used 40 years of community science data taken at 13 sites and in two seasons to assess population trends and relationships with climate variables (precipitation and temperature). We found that the declines seen in Arizona reflect the general trends found for western US. The preceding winter precipitation is a driver of both the number of species in the fall and spring surveys and the total number of unique butterflies in the spring surveys. The number of unique butterflies counted in the fall surveys was more affected by summer monsoon precipitations. Managers and policy makers may use these results, combined with climate projections, to consider implementing management actions to help counteract butterfly declines, such as creating more refugia for butterflies across the landscape.

**Abstract:**

Butterfly populations are declining worldwide, reflecting our current global biodiversity crisis. Because butterflies are a popular and accurate indicator of insect populations, these declines reflect an even more widespread threat to insects and the food webs upon which they rely. As small ectotherms, insects have a narrow range of habitable conditions; hence, extreme fluctuations and shifts caused by climate change may increase insects’ risk of extinction. We evaluated trends of butterfly richness and abundance and their relationship with relevant climate variables in Arizona, U.S.A., using the past 40 years of community science data. We focused on precipitation and temperature as they are known to be influential for insect survival, particularly in arid areas like southwestern U.S.A. We found that preceding winter precipitation is a driver of both spring and summer/fall butterfly richness and spring butterfly abundance. In contrast, summer/fall butterfly abundance was driven by summer monsoon precipitations. The statistically significant declines over the 40-year period were summer/fall butterfly abundance and spring butterfly richness. When controlling for the other variables in the model, there was an average annual 1.81% decline in summer/fall season butterfly abundance and an average annual decline of 2.13 species in the spring season. As climate change continues to negatively impact winter precipitation patterns in this arid region, we anticipate the loss of butterfly species in this region and must consider individual butterfly species trends and additional management and conservation needs.

## 1. Introduction

In the era of the Anthropocene, considered the sixth mass extinction, the biodiversity of insects is threatened [1,2]. Possibly 40% of insect species may face extinction in the coming decades, based on the current rates of declines [2], impacting critical ecosystem services such as pollination, nutrient cycling, and biological control [3,4]. Butterflies are experiencing comparable declines to all insects, with 8–29% of butterfly species extinct in European countries [5]. Certainly, habitat loss and changing agricultural practices have contributed to these patterns of decline [2], but the observed insect declines in many protected areas suggest climate change as a driver of this decline, as patterns of precipitation and temperature are also contributing to these population trends [6,7].

Climate change is having heterogeneous effects on various abiotic and biotic drivers of insect abundance and richness, which, in turn, influence insect declines [8,9]. Changes in abiotic drivers, such as precipitation and temperature, that have direct effects on butterflies can cause extirpations if pushed beyond the range of insect climate tolerance [10,11,12,13]. Researchers have recently found that, by explicitly modeling the regional differences in the distribution of temperatures projected with climate change, the dynamic and cumulative effects of thermal stress increased the extinction risk of ectotherms overall, but the trend was higher in the sub-tropics than in the tropics [8]. In areas where hot summer temperatures are already near the critical thermal maximum for a species, such as in hot deserts, an increase in temperatures will likely induce heat stress and reduce fitness for ectotherms [9]. Western butterflies in the United States have experienced about a ~2% decline a year across all species over the last 40 years [14]. These declines are correlated with increasing summer/fall temperatures and longer periods of aridity in the summer/fall, both attributes associated with climate change [14]. The biotic drivers of butterfly abundance and richness, such as food and nectar plant availability, are closely linked to environmental factors; thus, climate-induced changes, such as drought, which decrease plant abundance or specific host plants, can also threaten butterfly persistence [2,6].

The precipitation and temperature patterns of southwestern USA are distinct from the rest of western USA., with two rainy seasons (winter and summer) and extreme summer temperatures, followed by warm dry autumns. Although rainfall has increased in the past few years, the region is warming, and an increase in mean and extreme precipitation is predicted [15]. Climate change in arid regions does not always have intuitive effects on vegetation or soil moisture [16], leading to complex species responses. Thus, the influences of seasonal precipitation and temperature may have lagging effects not seen in other regions that only experience winter precipitation and more moderate summer temperatures. Given the distinctiveness of the climate in this region, we were interested in conducting a focused assessment of butterfly status in Arizona to investigate whether the trends found in western U.S. held true for this state. Specifically, we had the following objectives: (1) identify trends in butterfly abundance and richness over time in Arizona; and (2) investigate how climate variables drive butterfly abundance or richness in Arizona.

## 2. Methods

### 2.1. Study Sites

**Figure 1:**
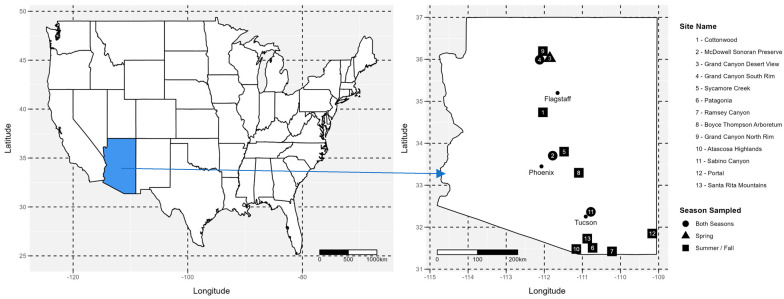
Location and sampling season of the 13 sites in Arizona, USA. The sites are labeled by numbers from the driest (1) to the wettest (13), based on the average annual precipitation (1991–2020; PRISM Climate Group, Oregon State University, https://prism.oregonstate.edu, data created 1 January 1981, accessed on 8 September 2022). The elevation ranges from 755 m to 2112 m. The shapes indicate the sampling season for each site: the circle indicates summer/fall; the square indicates spring; and the triangle indicates both spring and summer/fall.

**Figure 2:**
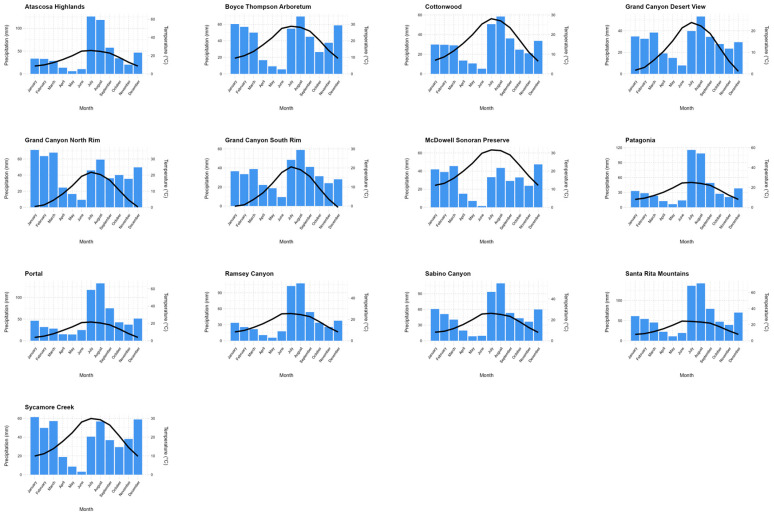
Trends of mean monsoon, winter, and annual precipitation over time for each of the 13 Arizona NABA survey sites. Each bar is the average monthly precipitation, and the black line is the average mean temperature by month for the study period (1981–2021) (PRISM Climate Group (Oregon State University, https://prism.oregonstate.edu, data created 1 January 1981, accessed on 8 September 2022).

For our analysis, we assembled the available data from 13 NABA sites in Arizona (Table 1 and Figure 1). The NABA sites range from 755 to 2112 m in elevation and cover the wide range of climates in Arizona (Table 1 and Table S1, and Figure 2). We started with the data assembled by Forister et al. (2021) that dated from 1979 to 2018. We updated these with the data for all sites through 2021 and added the data for the sites that had been omitted for not having 10 years of data (McDowell Sonoran Preserve, Cottonwood, Grand Canyon, Santa Rita; Table 1). Every study site experiences aspects of the North American monsoon phenomenon. It leads to a large increase in summer rainfall and a seasonal change in vegetation in southwestern United States and northwestern Mexico. In response, the butterfly communities in this area and their phenologies in the spring (pre-monsoon) and summer/fall (post-monsoon) seasons have different dynamics than those in areas outside the influence of the North American monsoon phenomenon. However, over the 40 years of the study period, the southwest has experienced long-term drought in the last 20 years [17].

### 2.2. Butterfly Surveys

The NABA data are based on citizen science surveys that follow procedures established by the North American Butterfly Association (NABA; https://naba.org/wp-content/uploads/2023/06/nabaus.pdf, accessed on 10 September 2022). Each site is a 24.14 km (15-mile) diameter circle in which multiple surveys can be conducted. The project leads submit report results to the NABA including the following: time, date, names of surveyors, weather conditions at the start and end of surveys, number of participants, number of parties, number of miles surveyed, number of party hours, and the count of each butterfly species observed. A party hour is defined as a group on a survey that spends one hour in the field actively butterflying on foot. The party hours were not constant across the sites or across the years, due to the citizen science data collection approach (Figure S1. The butterfly names were cleaned to be consistent with the NABA regional species list and the NABA taxonomy (Table S2). We analyzed data by common name, following the NABA taxonomy. In order to correct for uneven usage of subspecies across sites, we changed the common names so that species and subspecies were collapsed in the data. We also used common names to correct for changes in taxonomy not reflected on the NABA list. By changing only the common names we were able to preserve the Latin name as reported in the data. Finally, three species in the data were not listed in the NABA checklist but were checked and added (Table S2). Species richness and abundance were calculated from the counts for each sampling event. The butterflies that had not been identified as belonging to specific species were included in the total number of individuals (abundance) but were excluded from the total number of species (richness).

### 2.3. Environmental Data

We downloaded climate data for daily precipitation and temperature dating back to 1981 for each site from PRISM (PRISM Climate Group (Oregon State University, https://prism.oregonstate.edu, data created 1 January 1981, accessed on 8 September 2022). Based on these data, we summarized seasonal climate variables for analytical purposes (Table S3).

Arizona NABA sampling events occur from early April through October, and some sites host two sampling events each year, likely meant to capture the two distinct post rainfall periods which occur in this region. However, the national NABA protocol encourages surveys to occur near July 4th. We separated the data into two data sets to align with butterfly seasonality: spring (through early summer, March 1 to June 1) and summer/summer/fall (July 15 to October 31). This also served to separate spring and summer/fall sampling events that occurred at a single site. Four sampling events in the data set were removed because they fell between the two specified time frames (6/20/1982 Atascosa Highlands, 6/24/1982 Ramsey Canyon, 7/5/2015 Grand Canyon North Rim, 7/7/1998 Sycamore Creek).

The spring data set contains 17 sampling events across four sites dating back to 2011 (Table S1). The summer/fall data set contains 182 sampling events across 12 sites dating back to 1981 (Table S1). For years 2011–2021, the Santa Rita Mountains site contained two sampling events per year in the summer/fall data set: in this case, only the sampling event closest to the July 4th date was included to remain consistent with the timing of the other sampling events in the summer/fall and the NABA protocol.

### 2.4. Variable Selection

All potential climate predictors listed in Table S3 were considered for inclusion in the model. Stepwise selection was used to determine the best set of predictors. To achieve this, we compared candidate models with different temperature and precipitation variables (e.g., survey day, 30/90/365-day variables) for the final model selection. The variables were selected for the final model based on statistical significance, specifically the partial F-statistic. All the variables selected for the final model were evaluated for potential multicollinearity using a variance inflation factor (VIF) and a correlation matrix. The potential impact of sampling effort on the counts is statistically controlled for in all the potential models by including party hours as a fixed effect, which is a good proxy for effort. Party hours account for sampling effort by acknowledging the number of separate parties and the time each party spent recording data.

### 2.5. Modeling

For all the models, a general linear model was used to estimate the fixed effects of the climate variables on butterfly abundance and richness. For the models including summer/fall data sets, we used a mixed model to explicitly account for the variability between and within each site (Figure S1A–F) by estimating a random intercept for each site and the covariance within each site. In contrast, the spring data set contained much fewer sampling events; therefore, the inclusion of a random effect for site was not feasible. Instead, we compared the models with and without the Grand Canyon sites, which only had one spring survey each. Each model included the fixed effects of year and the following climate pattern predictors: maximum and minimum temperature over the 30-day time period prior to a sampling event, monsoon precipitation, and winter precipitation. Additionally, each model statistically controlled for the effort in sampling collection by including the nuisance variable party hours as a fixed effect. To gain insight into which species account for the loss in species numbers, we compared all the species found in the baseline years of spring (first three years: 2011–2013) and fall (early five years: 2002–2006) surveys with those in the most recent three years (2019–2021) of data. The baseline years of spring data included only the Sabino Canyon site, which was compared with the Sabino Canyon and the McDowell Sonoran Preserve sites for the most recent three years of data (the Grand Canyon sites are excluded because they only reported data in 2015). The baseline years of summer/fall data were chosen to maximize the number of sites reporting data early in the 40-year time span and includes five sites out of twelve (Ramsey Canyon, Patagonia, Portal, Sabino Canyon, and Boyce Thompson Arboretum; Table S1). We excluded Sycamore Creek because their surveys did not continue past 2004. Due to the uneven sampling across years and sites, this is an imperfect comparison and should be cautiously interpreted. To supplement this comparison, we checked the presence of species dropped in one season in the last three years of the data with the other season in the most recent three years of the data (Table S2).

In both the summer/fall and spring models, butterfly abundance was transformed using a logarithmic scale to normalize the response values for analysis. The summer/fall model for species richness utilized a square root transformation to stabilize the variance and satisfy the assumption of homogeneity of variances. All the analyses were performed with the R version 4.1.1 (R Core Team, 2021) with the use of the lme4 package [18]. All the figures were created in R version 4.1.1 [19].

## 3. Results

### 3.1. Butterfly Abundance and Richness Declines over Time and Observer Effort

**Figure 3:**
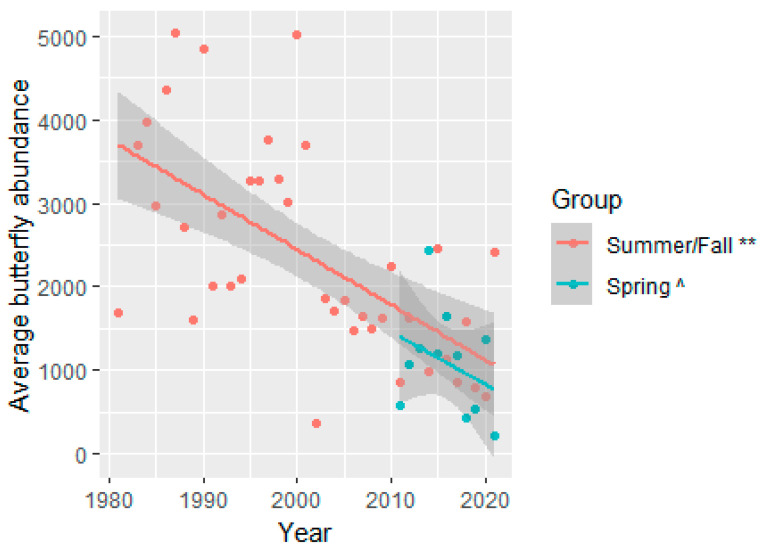
The average number of unique butterflies (mean butterfly abundance) shows a marginal decline in spring and a significant decline in summer/fall surveys over time, averaged over the other variables in the model. The lines represent linear model trend lines with the shaded areas representing 95% confidence intervals. The model included the following fixed factors: year, minimum and maximum temperatures, winter and summer monsoon precipitation, and party hours, with site as a random effect. Spring survey’s mean butterfly abundance was averaged across seventeen spring surveys conducted at four sites (blue line). Summer/fall survey’s mean abundance was averaged across 183 summer/fall surveys conducted at 12 sites (orange line). The symbols in the legend represent significance at the following levels: ** = *p* < 0.01; and ^ = *p* < 0.10.

**Figure 4:**
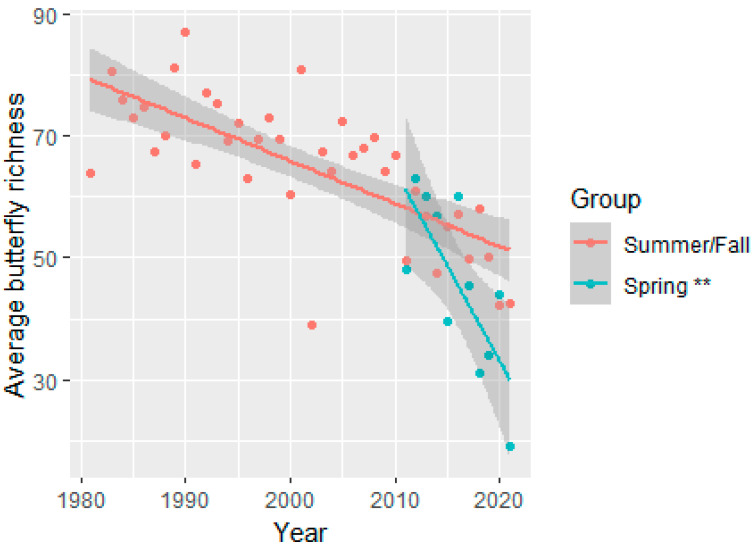
The total of unique butterfly species (mean butterfly richness) showed a significant decline in spring but not summer/fall season surveys, averaged over the other variables in the model. The lines represent linear model trend lines with the shaded areas representing 95% confidence intervals. The model included the following fixed factors: year, minimum and maximum temperatures, winter and summer monsoon precipitation, and party hours, with site as a random effect. Spring survey’s mean butterfly richness was averaged across seventeen spring surveys conducted at four sites (blue line). Summer/fall survey’s mean richness was averaged across 183 summer/fall surveys conducted at 12 sites (orange line). Each point represents the average of all the surveys taken within that season/year. The asterisks represent significance at the following levels: ** = *p* < 0.01.

Butterfly abundance declined over the 40-year sampling period for summer/fall surveys at an average annual rate of 1.81% when controlling for the other variables in the model (summer/fall abundance model, year: t_(df=143.8)_ = −2.725, *p* = 0.0072, Figure 3 and Table S4). The decline in summer/fall season butterfly richness over time was negligible and not significant. Butterflies had a different pattern in the spring: butterfly abundance declines were only marginally significant (at α = 0.10 level) at an average annual rate of 9.49% attributable to the fixed factor year when controlling for the other variables in the model (spring abundance model, year: (t_(df=10)_ = −1.836, *p* = 0.0962, Table S5 and Figure 3). The butterfly richness, for the spring surveys, declined significantly, at an average rate of 2.13 individual species per year, when controlling for the other variables in the model (spring richness model: (t_(df=10)_ = −3.803, *p* = 0.0036, Figure 4 and Table S6). Compared with the spring baseline, eleven species were missing in the spring surveys, but all of these were seen in the summer/fall season in the most recent three years (2019–2021, Table S2). Compared with the fall baseline, 13 species were not present in the most recent three years (2011–2013) of fall but were also not observed in the spring surveys (Table S2). The 13 species that had dropped in recent years were in the following families (number of species missing): Hesperiidae (7), Lycaenidae (1), Nymphalidae (3), and Pieridae (2; Table S2). As noted in the methods section, these findings should be considered with caution, because not all surveys were included in the beginning and end of these comparisons, and 81 species (30.4% of all the species in the data) were not seen in either the baseline years or the most recent three years (Table S2).

Observer effort was a strong predictor of both butterfly richness and abundance. Party hours, our proxy for effort, were highly significant for butterfly abundance and richness (*p* < 0.001 in all four models, Tables S4–S7). This suggests that the more time observers spent in the field, the larger the values for both butterfly abundance and richness. We note that the strong relationship between abundance and richness and the number of party hours occurred at the site level (Figure S1A–F) and over time (Figure S2G,H). The final model estimates presented in Tables S2–S6 include party hours, and, thus, other estimates are statistically adjusted for the effect of observer effort. The VIF was tested with a result close to one for the summer/fall richness and abundance model variables and closer to two in the spring abundance and richness models, suggesting no or low multicollinearity (Table S8).

### 3.2. Effect of Climate Patterns on Butterfly Abundance

**Figure 5:**
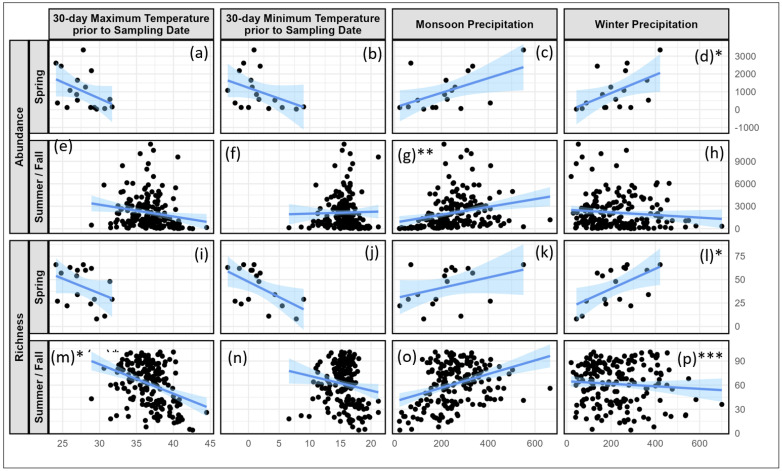
The maximum and minimum temperatures for the preceding 30 days and seasonal precipitation had significant effects on the abundance and number of unique butterflies, averaged over the other variables in the model. Each point represents the average butterfly (Y-axis) and climate (X-axis) metric at each site. The lines represent linear model trend lines with the shaded areas representing 95% confidence intervals. The model included the following fixed factors: year, minimum and maximum temperatures, winter and summer monsoon precipitation, and party hours, with site as a random effect. Effect of the max temperatures within 30 days prior to the sampling date of the survey on spring survey butterfly (**a**) abundance and (**i**) richness and summer/fall survey (**e**) abundance and (**m**) richness. Effect of the minimum temperature within 30 days prior to the sampling date of the survey on spring survey butterfly (**b**) abundance and (**j**) richness and summer/fall survey (**f**) abundance and (**n**) richness. Monsoon precipitation effect on spring survey butterfly (**c**) abundance and (**k**) richness and summer/fall survey (**g**) abundance and (**o**) richness. Winter precipitation effect on spring survey butterfly (**d**) abundance and (**l**) richness and summer/fall survey (**h**) abundance and (**p**) richness. Precipitation is shown in mm and temperatures in degrees Celsius. The asterisks (positioned in the figures, next to the letters) represent significance at the following levels: *** = *p* < 0.001; ** = *p* < 0.01; and * = *p* < 0.05.

The previous season’s monsoon but not winter precipitation significantly contributes to the summer/fall abundance of butterflies (monsoon precipitation: t_(df=173.693)_ = 3.438, *p* = 0.0007; winter precipitation: t_(df=172.885)_ = −0.097, *p* = 0.9227, Figure 5g,h and Table S4). Similarly, the temperature predictors, i.e., previous 30-day min and max, did not significantly impact summer/fall butterfly abundance (min temp: t_(df=79.982)_ = 0.821, *p* = 0.4140, Table S4, Figure 5f; and max temp: t_(df=168.141)_ = −0.777, *p* = 0.4384, respectively, Figure 5e and Table S4). The model estimates that a 1 cm decrease in monsoon precipitation results in a 2.4% decrease in summer/fall season butterfly abundance, when controlling for the other variables in the model.

In contrast, winter precipitation significantly contributes to butterfly abundance in the spring model, while monsoon precipitation does not (winter precipitation: t_(df=10)_ = 2.9703, *p* = 0.0007; monsoon precipitation: t_(df=10)_ = −0.152, *p* = 0.8821, Figure 5c,d and Table S5). Similar to the model for the summer/fall sampling events, temperature predictors were insignificant in the model (Figure 5a,b). The model estimates that every 1 cm decrease in winter precipitation results in a 3.7% decrease in spring season butterfly abundance, when controlling for the other variables in the model.

Since we were not able to include site as a random effect and the Grand Canyon sites had only one observation each, we analyzed the spring models with and without those two sites. With the sites excluded, the effect of party hours was less significant (t_(df=8)_ = 3.268, *p* = 0.0114), but the difference between models for the other fixed effects was minimal. Since the exclusion did not dramatically change the output of the model, we reported results with all sites included in the model.

### 3.3. Effect of Climate Patterns on Butterfly Richness

The previous season’s winter precipitation significantly contributed to spring richness of butterflies, while the most recent monsoon precipitation was marginally significant (winter: t_(df=10)_ = 2.784, *p* = 0.0193; monsoon: t_(df=10)_ = −2.162, *p* = 0.0562, Figure 5k–l and Table S6). The spring richness model estimates indicate that a decrease of 2.364 cm in winter precipitation results in a loss of one butterfly species, when controlling for the other variables in the model. The previous 30-day min and max temperature predictors did not significantly impact the richness of butterflies (min temp: (t_(df=10)_ = −1.426, *p* = 0.1843; max temp: t_(df=10)_ = −0.113, *p* = 0.9122, Figure 5i–j and Table S7).

As in the spring butterfly abundance model, we analyzed the spring richness model with and without the Grand Canyon sites. Without these sites included, the effects of year and party hours were still significant but less so (year: t_(df=8)_ = −2.413, *p* = 0.0423; party hours: t_(df=8)_ = 2.991, *p* = 0.0173), and the differences between the models for the other fixed effects were not significant. Since the exclusion did not dramatically change the output of the model, we reported results with all sites included in the model.

The summer/fall model also estimated that the previous season’s winter but not monsoon precipitation significantly contributed to butterfly richness (winter precip: t_(df=165.3)_ = 3.294, *p* = 0.0012; monsoon precip: t_(df=169.38)_ = 0.903, *p* = 0.3680, Figure 5o–p and Table S7). While this is statistically significant, it is not ecologically significant, as it is estimated that a decrease of 62.5 cm in winter precipitation is required to result in a loss of one butterfly species, when controlling for the other variables in the model. The previous 30-day maximum temperature also significantly impacted butterfly richness in the summer/fall season (max temp: t_(df=172.3)_ = −2.011, *p* = 0.0459, Figure 5m and Table S7), while the minimum temperature did not (min temp: t_(df=163.6)_ = 0.309, *p* = 0.7574, Figure 5n and Table S7).

## 4. Discussion

Here, we found decreases in butterfly abundance and richness through time across all sites and seasons controlling for party hours. These trends align broadly with the butterfly declines observed in other butterfly studies in western USA [14]. However, the responses depended on seasonality. While spring season butterfly abundance declines were only marginally significant by year, spring season butterfly species richness showed highly significant declines over time when averaged over the other variables in the models. In contrast, for the summer/fall season, butterfly abundance declined significantly by year, and butterfly species richness appeared stable over time. When we looked at which species were missing from the last years of survey data, although eleven species were missing from the spring surveys, all these species were seen in the summer/fall surveys during that same time period. In contrast, of the 13 species missing from the summer/fall surveys, none were seen in recent spring surveys. The shift in butterflies from spring to summer or fall may be due to natural fluctuations in phenology combined with the short, one-day sampling events employed; others have highlighted the problem with short survey periods missing butterflies due to fluctuations in phenology [20]. Alternatively, these trends may be a cue of butterflies shifting or shortening their season in Arizona. Researchers have documented changes in butterfly phenology, driven by changes in temperature [21,22,23], and have detected differences in phenology between urban and rural areas [24].

Regarding the 13 species which were not seen in the summer/fall surveys during the last three years of survey data compared to the baseline years, there are multiple reasons why butterflies may no longer be seen at a particular site. Foremost, detection can be a challenge with any monitoring approach. In particular, skippers, representing 54% of the missing species, can be hard to detect and identify by many observers. Other biological mechanisms that could be at play are the following: increased aridity leading to decreases in hostplant availability, vagrant species (i.e., soldier), or eruptive species (i.e., Mexican fritillary). But, it was beyond the scope of this work to identify drivers of species losses. This highlights the need for ongoing long-term monitoring and for additional work focusing on individual species trajectories and mechanisms of decline.

We found that, of the climate variables we compared, winter precipitation most affected patterns of butterfly abundance and richness across seasons. The butterfly richness of both the summer/fall and spring surveys depended on winter rains, but abundance depended on the most recent rains (spring surveys depended on winter rains, and summer/fall survey abundance depended on monsoon rains). This is an interesting finding because it shows that, while the number of butterflies responds to recent rains (whether winter or monsoon) and subsequent green-up and flowering, species richness is governed by winter rains, and certain species may not appear in the region following winter drought.

Over the past 40 years, Arizona has experienced long-term drought, and precipitation has declined across our sites. However, recent models predict that, by the end of this century, extreme wet and dry events will increase in the region, with a greater frequency in wet extreme events and an overall state-wide increase in winter rains [15], which may be good news for the stability of Arizona butterfly populations. However, depending on species-specific tolerances, some butterfly species may not be able to survive extreme and/or prolonged drought or increased heat [25], and some specialist species are likely to be more affected than generalist ones depending on the availability of their hostplants [26]. Since butterflies are herbivores, drought impacts are largely mediated by plants, and drought can cause changes to plants’ secondary defense chemistry and possibly alter host plant palatability, with unknown ecosystem effects if droughts persist [27].

Temperature (high max temperatures for the previous 30 days) was significant only in the summer/fall richness model and not in the other models. Unlike our study, others have found temperature to result in the most frequent effects on butterflies, compared with other weather variables [26]. The fact that, in our study, temperature affected richness and not abundance indicates that some species may drop out in response to high heat. As ectotherms, butterflies are sensitive to temperatures and stressful temperatures can increase mortality [9,28], but the effect of extreme heat on butterflies can depend on their life stage [29]. Similarly, a study across 84 locations in Germany found that, although insect biomass increases linearly with temperature, this trend reversed when local averages exceeded long-term averages in the summer [30]. Rather than a loss of species (extinctions), butterflies in Arizona may be responding to changes in the climate by shifting their range. In a study of nonmigratory European butterflies, 35 of the 57 species studies had undergone range shifts [31].

Along with the interannual fluctuations linked to precipitation and, to a lesser extent, temperature, other stressors contributing to the long-term decline shown over the 40-year span of the butterfly data utilized in this study may have been widespread habitat conversion across Arizona [32,33,34] and long-term drought [35,36]. A review of butterfly declines and their causes in Europe indicates extinctions from 8 to 29% and declines in abundances of 30–50%, with 82 species listed as threatened or near-threatened on the IUCN Red List [5]. Degradation and loss of habitat and chemical pollution were thought to be the main causes of this decline [5]. Butterflies are sensitive to habitat loss at the patch and landscape scale [37].

## 5. Conclusions

Since trends in butterfly populations are good indicators for insect populations [24,38,39], the broader implications for insects and the food webs dependent on insects do not paint a rosy picture. Conservation measures targeted at supporting insect populations should strive to maintain a large habitat patch size, good patch quality, reduce patch isolation [40], and reduce climate change to allow for maximum time for species adaptation [25]. In addition, ensuring reserves that can provide microclimates and refugia, such as those found in botanical gardens, may also help mitigate against the effects of climate change [41].

## Figures and Tables

**Table 1:** Long-term mean climate variables for spring (March 1–June 15) and summer/fall (July 15–October 31) temperatures and annual precipitation (1991–2020). The sites are ordered from the driest to the wettest, based on the average annual precipitation. The standard deviation is shown in parentheses.

Sites	Average Spring Min Temp (°C)	Average Spring Max Temp (°C)	Average Summer/Fall Min Temp (°C)	Average Summer/Fall Max Temp (°C)	Average Annual Precipitation (mm)
1. Cottonwood	7.28 (4.74)	26.45 (6.84)	14.67 (5.24)	32.60 (5.52)	328.73 (85.13)
2. McDowell Sonoran Preserve	14.97 (5.25)	27.61 (6.43)	22.82 (4.43)	34.50 (5.01)	332.18 (128.28)
3. Grand Canyon Desert View	4.26 (5.64)	19.87 (7.03)	11.32 (5.31)	26.64 (5.89)	340.72 (85.13)
4. Grand Canyon South Rim	−0.40 (5.06)	17.66 (7.05)	6.90 (5.17)	24.65 (5.73)	372.68 (95.20)
5. Sycamore Creek	11.95 (5.24)	26.70 (6.65)	19.63 (4.52)	33.26 (5.17)	433.27 (183.62)
7. Ramsey Canyon	8.97 (4.98)	25.82 (6.03)	15.14 (4.09)	29.69 (4.09)	458.69 (116.32)
8. Boyce Thompson Arboretum	12.06 (5.32)	25.78 (6.48)	19.21 (4.33)	31.99 (4.75)	463.56 (185.35)
9. Grand Canyon North Rim	2.56 (5.65)	17.59 (7.12)	9.61 (5.16)	24.66 (5.95)	498.12 (148.32)
10. Atascosa Highlands	8.32 (4.78)	25.92 (5.95)	15.17 (4.22)	30.05 (3.95)	499.72 (108.85)
11. Sabino Canyon	9.67 (5.34)	24.68 (6.42)	16.55 (4.04)	29.84 (4.39)	552.50 (190.40)
12. Portal	4.85 (4.98)	21.60 (6.00)	11.17 (4.21)	25.47 (4.01)	585.46 (149.55)
13. Santa Rita Mountains	9.24 (5.28)	23.60 (6.32)	15.65 (3.71)	27.48 (4.16)	657.68 (175.22)

## Data Availability

Raw data and analysis scripts are available at the GitHub Repo: https://github.com/jenbroatch/AZButterFlyDiversity/tree/main, accessed on 11 November 2023.

## Supplementary Materials

The following supporting information can be downloaded at: https://www.mdpi.com/article/10.3390/insects15010005/s1. The following are also available online on GitHub: https://github.com/jenbroatch/AZButterFlyDiversity/tree/main. Supplementary Table S1. Survey site location, elevation, timing, and years sampled. Supplementary Figure S1 Distribution of mean butterfly abundance, richness, and party hours by site for seasonal surveys. Box plots show the (A) butterfly abundance, (B) butterfly richness, and (C) party hours for the sites that conducted the summer/fall surveys averaged over time and (D) butterfly abundance and (E) butterfly richness, and (F) party hours for the sites that conducted the spring surveys averaged over time, and the party hours of the spring surveys (G) and summer/fall surveys (H) over time, averaged over the NABA survey sites. Supplementary Table S2. List of butterfly species seen in our surveys. Supplementary Table S3. Climate variables and descriptions considered for inclusion in the final model. Supplementary Table S4. Linear mixed model output table for summer/fall survey butterfly abundance. Supplementary Table S5. General linear model output table for spring survey butterfly abundance. Supplementary Table S6. General mixed model output table for spring survey butterfly richness. Supplementary Table S7 Linear mixed model output table for summer/fall survey butterfly richness. Supplementary Table S8. Variance inflation factor (VIF) output for each model.
