# Supplementary material for: Winter Rains Support Butterfly Diversity, but Summer Monsoon Rainfall Drives Post-Monsoon Butterfly Abundance in the Arid Southwest of the US
Source: Insects. 2023 Dec 21;15(1):5. doi: 10.3390/insects15010005 (PMC10816195; doi:10.3390/insects15010005)
Supplement: Supplementary file 1 [file insects-15-00005-s001.zip › insects-2732156-supplementary.pdf]

Table S1: Survey site location, elevation, timing and years sampled.

Site	Location (°W, °N)	Elevation (m)	Sampling Season	Sampling Year(s)
Atascosa Highlands	31.4800, -111.1600	1353	Summer/Fall	1983-2001
Boyce Thompson Arboretum	33.2950, -111.0946	1087	Summer/Fall	2007-2021
Cottonwood	34.7380, -112.0353	1071	Summer/Fall	2017-2021
Grand Canyon Desert View	36.0244, -111.8554	1832	Spring	2015
Grand Canyon North Rim	36.1954, -112.0374	2112	Summer/Fall	2019 & 2021
Grand Canyon South Rim	35.9890, -112.1221	2061	Spring	2015
			Summer/Fall	2014-2015
McDowell Sonoran Preserve	33.7041, -111.7791	755	Spring	2017-2021
			Summer/Fall	2014-2021
Patagonia	31.5073, -110.7331	1434	Summer/Fall	1986-2021
Portal	31.8500, -109.1667	2000	Summer/Fall	1999-2005 & 2009 & 2012-2021
Ramsey Canyon	31.4240, -110.2238	1460	Summer/Fall	1981 & 1983-2021
Sabino Canyon	32.3656, -110.7774	1484	Spring	2011-2018 & 2020-2021
			Summer/Fall	1991-1998 & 2000-2002 & 2008- 2021
Santa Rita Mountains	31.7268, -110.8723	1786	Summer/Fall	2011-2021
Sycamore Creek	33.8000, -111.4800	958	Summer/Fall	1999-2000 & 2004

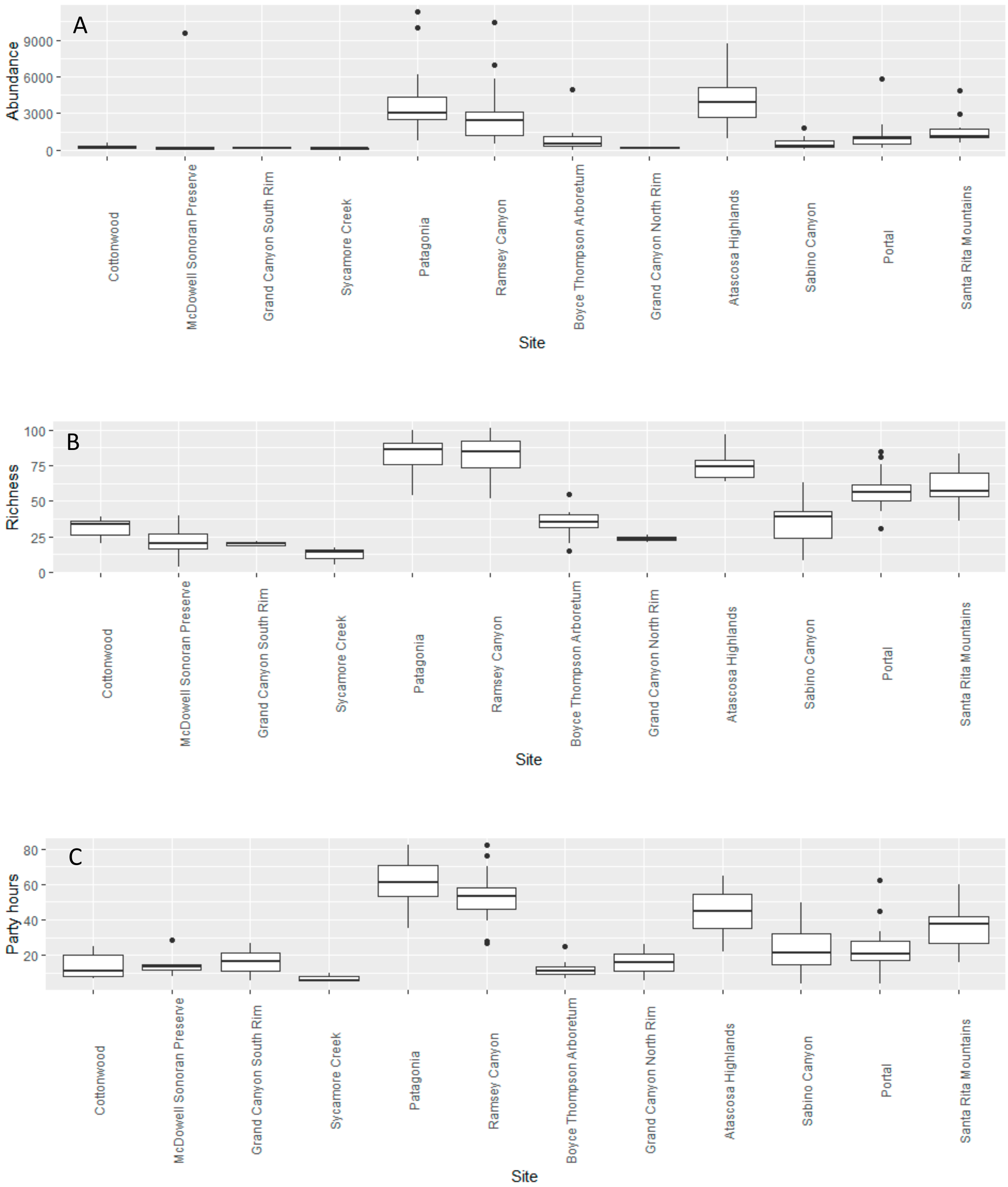
A
Abundance
9000
6000
3000
0
Cottonwood
McDowell Sonoran Preserve
Grand Canyon South Rim
Sycamore Creek
Patagonia
Ramsey Canyon
Boyce Thompson Arboretum
Grand Canyon North Rim
Atascosa Highlands
Sabino Canyon
Portal
Santa Rita Mountains
Site
B
Richness
100
75
50
25
0
Cottonwood
McDowell Sonoran Preserve
Grand Canyon South Rim
Sycamore Creek
Patagonia
Ramsey Canyon
Boyce Thompson Arboretum
Grand Canyon North Rim
Atascosa Highlands
Sabino Canyon
Portal
Santa Rita Mountains
Site
C
Party hours
80
60
40
20
Cottonwood
McDowell Sonoran Preserve
Grand Canyon South Rim
Sycamore Creek
Patagonia
Ramsey Canyon
Boyce Thompson Arboretum
Grand Canyon North Rim
Atascosa Highlands
Sabino Canyon
Portal
Santa Rita Mountains
Site

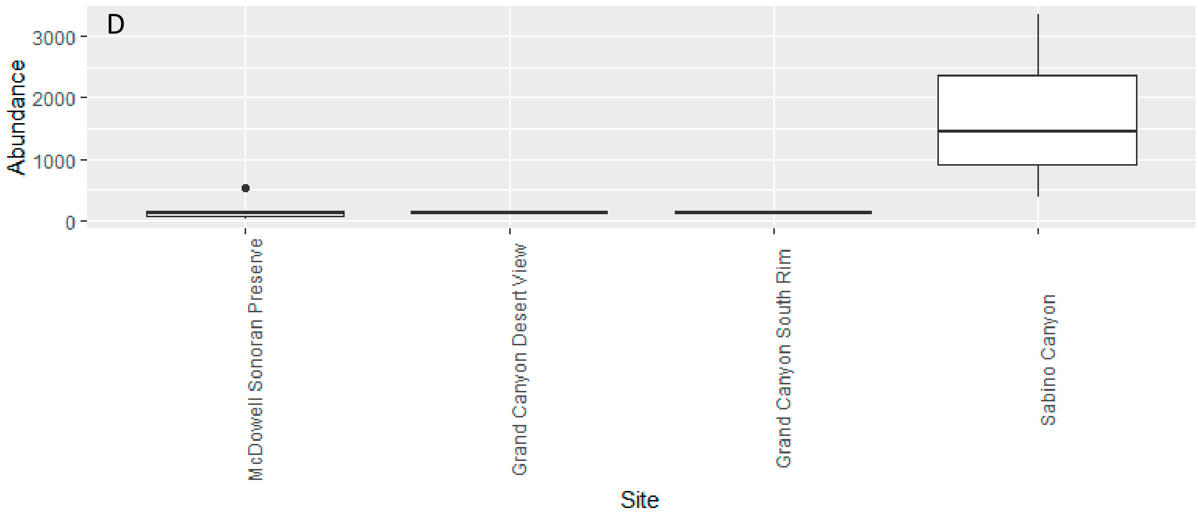
D
Abundance
3000
2000
1000
0
McDowell Sonoran Preserve
Grand Canyon Desert View
Grand Canyon South Rim
Sabino Canyon
Site

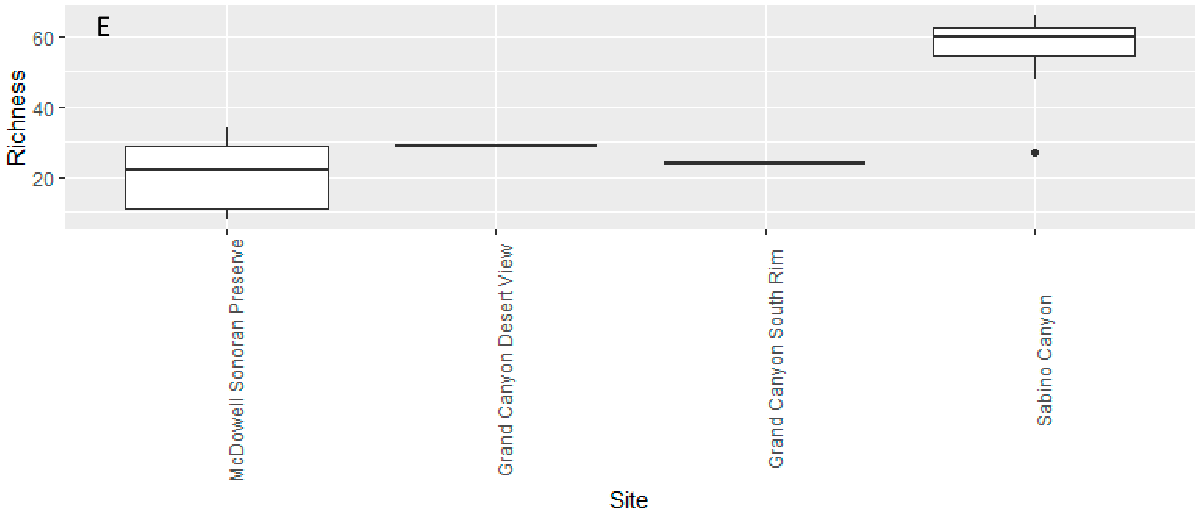
E
Richness
60
40
20
McDowell Sonoran Preserve
Grand Canyon Desert View
Grand Canyon South Rim
Sabino Canyon
Site

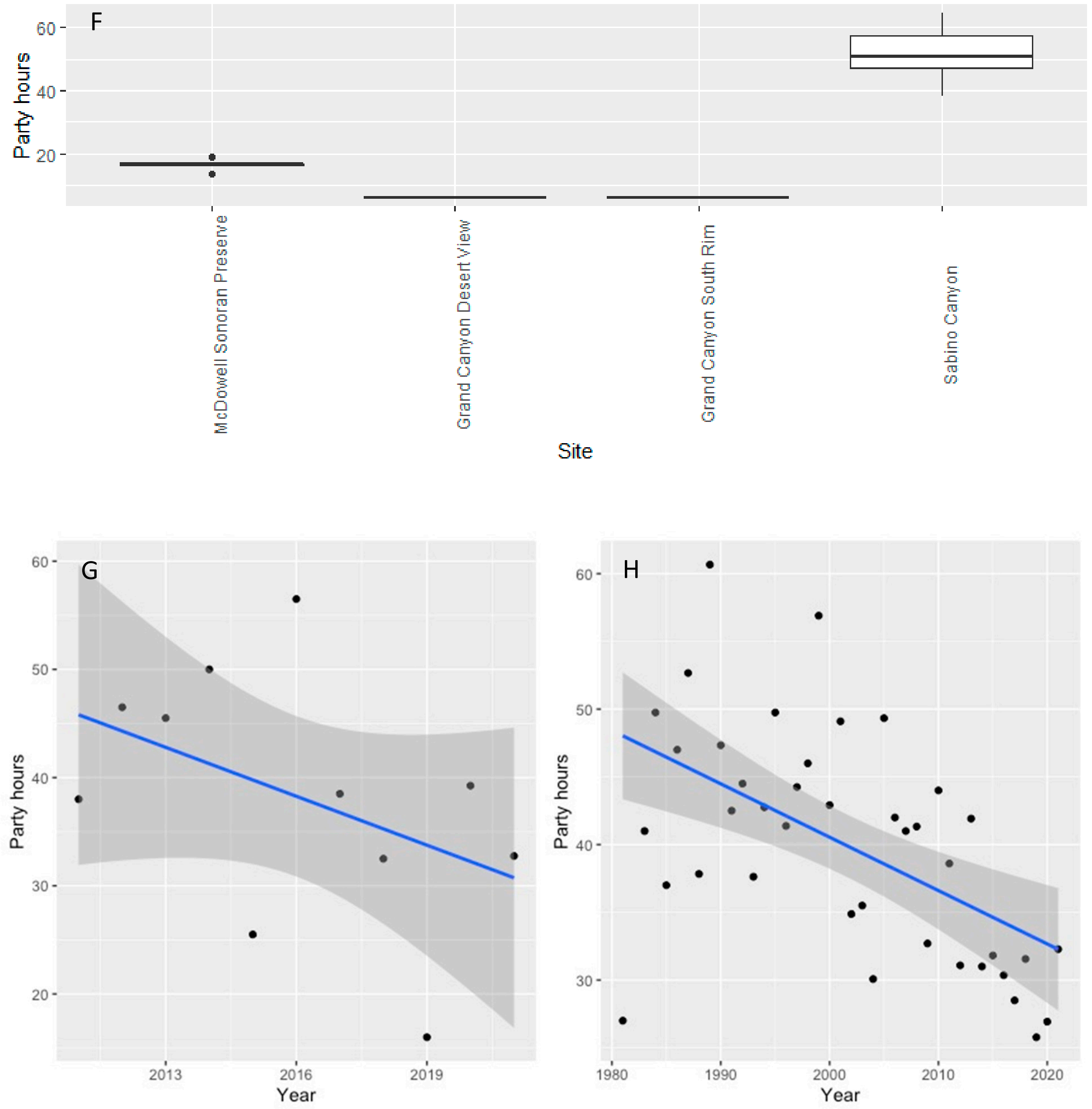

Figure S1. Distribution of mean butterfly abundance, richness, and party hours by site for seasonal surveys. Box plots show the (A) butterfly abundance, (B) butterfly richness, and (C) party hours for sites that conducted the summer/fall surveys averaged over time and (D) butterfly abundance and (E) butterfly richness, and (F) party hours of for sites that conducted spring surveys averaged over time, and party hours of (G) spring surveys and (H) summer/fall surveys over time, averaged over NABA survey sites. Sites in figures A-F are ordered left to right from driest to wettest based on the average annual precipitation for each site (Table 1). Summer/fall survey sites were included as random effects in the models to account for the different distributions. For the spring surveys, due to low sample size, they were not included as random effects, but models were compared with and without different sites instead. Party hours are calculated as the sum of the number of hours for each party on a single survey within site.

Party hours were included as fixed effects in all models. For plots A-F, boxes represent 50% of the values, the center line of the box represents the median value, each whisker represents 25% of the values, and dots are outliers. For plots G & H, each point represents the average value of all sites within that season/year. The lines represent trend lines with the shaded areas representing 95% confidence intervals.

Table S2. List of butterfly species seen in our surveys. The list follows NABA taxonomy for the southwestern United States. Species in bold were not on the original NABA taxonomy list, but added because they were observed in the NABA surveys and known to occur in this region. We collapsed subspecies to species in our analysis. An asterisk indicates instances of name changes and how they were collapsed. Season missing indicates that the species has not been seen in the last three years of surveys (2019-2021) within season indicated compared with baseline years (2011-2013 for spring surveys and 2002-2006 for fall surveys) across sites. A dash indicates that the species did not occur during the baseline or was not missing. Season seen indicates whether the species was seen between 2019-2021 and specifies the season seen. Species highlighted in red missing in years 2019-2021 compared to baseline within season and were also not seen in the other season in recent years (2019-2021). A dash indicates that the species was not seen during the 2019-2021 period (a dash in the season missing column and in the season seen column indicates that the species did not occur at all in the baseline and recent years).

**Family**	**LatinAnalysisNameCorrected**	**NABAEnglishName**	**Season missing**	**Season seen**
Hesperiidae	Achalarus_casica	desert cloudywing	Spring	Summer/Fall
Hesperiidae	Adopaeoides_prittwitzi	sunrise skipper	-	-
Hesperiidae	Agathymus_aryxna	arizona giant skipper	-	Summer/Fall
Hesperiidae	Amblyscirtes_aenus	bronze roadside skipper	-	Summer/Fall
Hesperiidae	Amblyscirtes_cassus	cassus roadside skipper	-	Summer/Fall
Hesperiidae	Amblyscirtes_elissa	elissa roadside skipper	-	Summer/Fall
Hesperiidae	Amblyscirtes_eos	dotted roadside skipper	Spring	Summer/Fall
Hesperiidae	Amblyscirtes_exoteria	large roadside skipper	-	Summer/Fall
Hesperiidae	Amblyscirtes_fimbriata	orange edged roadside skipper	-	Summer/Fall
Hesperiidae	Amblyscirtes_nereus	slaty roadside skipper	-	Summer/Fall
Hesperiidae	Amblyscirtes_nysa	nysa roadside skipper	-	Summer/Fall
Hesperiidae	Amblyscirtes_oslari	oslar's roadside skipper	-	Summer/Fall
Hesperiidae	Amblyscirtes_phylace	orange headed roadside skipper	Summer/ Fall	-
Hesperiidae	Amblyscirtes_simius	simius roadside skipper	-	-
Hesperiidae	Amblyscirtes_texanae	texas roadside skipper	-	Summer/Fall
Hesperiidae	Amblyscirtes_tolteca	toltec roadside skipper	-	Summer/Fall

Hesperiidae	Ancyloxypha_arene	tropical least skipper	-	Summer/Fall
Hesperiidae	Atalopedes_campestris	sachem	-	Summer/Fall
Hesperiidae	Atrytonopsis_cestus	cestus skipper	-	-
Hesperiidae	Atrytonopsis_edwardsii	sheep skipper	Spring	Summer/Fall
Hesperiidae	Atrytonopsis_lunus	moon marked skipper	-	Summer/Fall
Hesperiidae	Atrytonopsis_pittacus	white barred skipper	-	Spring&-
Hesperiidae	Atrytonopsis_python	python skipper	-	-
Hesperiidae	Autochton_cellus	golden banded skipper	-	Summer/Fall
Hesperiidae	Calpodes_ethlius	brazilian skipper	Summer/ Fall	-
Hesperiidae	Celotes_nessus	common streaky skipper	-	Spring&Summer/Fall
Hesperiidae	Chioides_catillus	white striped longtail	Spring	Summer/Fall
Hesperiidae	Chiomara_asychis	white patched skipper	-	Spring&Summer/Fall
Hesperiidae	Codatractus_arizonensis	arizona skipper	-	Summer/Fall
Hesperiidae	Codatractus_mysie	valeriana skipper	-	Summer/Fall
Hesperiidae	Cogia_caicus	gold costa skipper	-	Summer/Fall
Hesperiidae	Cogia_hippalus	acacia skipper	Spring	Summer/Fall
Hesperiidae	Copaeodes_aurantiacus	orange skipperling	-	Spring&Summer/Fall
Hesperiidae	Copaeodes_minimus	southern skipperling	-	Summer/Fall
Hesperiidae	Epargyreus_clarus	silver spotted skipper	-	Spring&Summer/Fall
Hesperiidae	Erynnis_afranius	afranius duskywing	-	Summer/Fall
Hesperiidae	Erynnis_brizo	sleepy duskywing	-	Spring&-
Hesperiidae	Erynnis_funeralis	funereal duskywing	-	Spring&Summer/Fall
Hesperiidae	Erynnis_icelus	dreamy duskywing	-	-
Hesperiidae	Erynnis_juvenalis	juvenal's duskywing	Spring	Summer/Fall
Hesperiidae	Erynnis_meridianus	meridian duskywing	Spring	Summer/Fall
Hesperiidae	Erynnis_pacuvius	pacuvius duskywing	-	Spring&Summer/Fall
Hesperiidae	Erynnis_scudderi	scudder's duskywing	Summer/ Fall	-
Hesperiidae	Erynnis_telemachus	rocky mountain duskywing	-	-
Hesperiidae	Erynnis_tristis	mournful duskywing	-	Spring&Summer/Fall
Hesperiidae	Euphyes_vestris	dun skipper	-	Summer/Fall

Hesperiidae	Heliopetes_domicella	erichson's white skipper	-	Spring&Summer/Fall
Hesperiidae	Heliopetes_ericetorum	northern white skipper	-	Summer/Fall
Hesperiidae	Heliopetes_laviana	laviana white skipper	-	Summer/Fall
Hesperiidae	Hesperia_comma	common branded skipper	Summer/Fall	-
Hesperiidae	Hesperia_pahaska	pahaska skipper	-	Spring&Summer/Fall
Hesperiidae	Hesperia_uncas	uncas skipper	-	-
Hesperiidae	Hesperopsis_alpheus	saltbush sootywing	-	Summer/Fall
Hesperiidae	Hylephila_phyleus	fiery skipper	-	Spring&Summer/Fall
Hesperiidae	Lerema_accius	clouded skipper	-	Summer/Fall
Hesperiidae	Lerodea_arabus	violet clouded skipper	-	Spring&Summer/Fall
Hesperiidae	Lerodea_eufala	eufala skipper	-	Spring&Summer/Fall
Hesperiidae	Megathymus_streckeri	strecker's giant skipper	-	-
Hesperiidae	Megathymus_ursus	ursine giant skipper	-	-
Hesperiidae	Oarisma_edwardsii	edwards skipperling	-	Summer/Fall
Hesperiidae	Oarisma_garita	garita skipperling	-	Summer/Fall
Hesperiidae	Ochlodes_sylvanoides	woodland skipper	-	Summer/Fall
Hesperiidae	Paratrytone_snowi	snow's skipper	-	Summer/Fall
Hesperiidae	Pholisora_catullus	common sootywing	-	Spring&Summer/Fall
Hesperiidae	Piruna_cingo	many spotted skipperling	-	Summer/Fall
Hesperiidae	Piruna_pirus	russet skipperling	-	-
Hesperiidae	Piruna_polingi	four spotted skipperling	-	Summer/Fall
Hesperiidae	Poanes_taxiles	taxiles skipper	-	Summer/Fall
Hesperiidae	Polites_carus	carus skipper	-	Summer/Fall
Hesperiidae	Polites_draco	draco skipper	-	Summer/Fall
Hesperiidae	Polites_themistocles	tawny edged skipper	-	-
Hesperiidae	Polygonus_leo	hammock skipper	-	Summer/Fall
Hesperiidae	Pyrgus_albescens	white checkered skipper	-	Spring&Summer/Fall
Hesperiidae	Pyrgus_communis	common checkered skipper	-	-
Hesperiidae	Pyrgus_oileus	tropical checkered skipper	-	-

Hesperiidae	Pyrgus_philetas	desert checkered skipper	-	Spring&Summer/Fall
Hesperiidae	Pyrgus_scriptura	small checkered skipper	Summer/Fall	-
Hesperiidae	Pyrrhopyge_araxes	dull firetip	-	Summer/Fall
Hesperiidae	Staphylus_ceos	golden headed scallopwing	-	Spring&Summer/Fall
Hesperiidae	Systasea_pulverulenta	texas powdered skipper	Summer/Fall	-
Hesperiidae	Systasea_zampa	arizona powdered skipper	-	Spring&Summer/Fall
Hesperiidae	Thorybes_drusius	drusius cloudywing	-	Summer/Fall
Hesperiidae	Thorybes_mexicanus	mexican cloudywing	-	-
Hesperiidae	Thorybes_pylades	northern cloudywing	-	Summer/Fall
Hesperiidae	Urbanus_dorantes	dorantes longtail	-	Summer/Fall
Hesperiidae	Urbanus_proteus	long tailed skipper	Summer/Fall	-
Hesperiidae	Zestusa_dorus	short tailed skipper	Spring	Summer/Fall
Lycaenidae	Agriades_glandon	arctic blue	-	-
Lycaenidae	Atlides_halesus	great purple hairstreak	-	Spring&Summer/Fall
Lycaenidae	Brephidium_exile	western pygmy blue	-	Spring&Summer/Fall
Lycaenidae	Callophrys_augustinus	brown elfin	-	Spring&-
Lycaenidae	Callophrys_dumetorum	canyon bramble hairstreak	-	-
Lycaenidae	Callophrys_gryneus	juniper hairstreak	-	Spring&Summer/Fall
Lycaenidae	Callophrys_spinetorum	thicket hairstreak	-	Spring&Summer/Fall
Lycaenidae	Callophrys_xami	xami hairstreak	-	-
Lycaenidae	Celastrina_ladon	spring azure	-	Spring&Summer/Fall
Lycaenidae	Erora_quaderna	arizona hairstreak	-	Spring&Summer/Fall
Lycaenidae	Euphilotes_battoides	square spotted blue	-	Spring&-
Lycaenidae	Euphilotes_rita	rita blue	-	-
Lycaenidae	Euphilotes_spaldingi	spalding's blue	-	Summer/Fall
Lycaenidae	Everes_amyntula	western tailed blue	-	Summer/Fall
Lycaenidae	Everes_comyntas	eastern tailed blue	-	-
Lycaenidae	Glaucopsyche_lygdamus	silvery blue	-	-

Lycaenidae	Hemiargus_ceraunus	ceraunus blue	-	Spring&Summer/Fall
Lycaenidae	Hemiargus_isola	reakirt's blue	-	Spring&Summer/Fall
Lycaenidae	Hypaurotis_crysalus	colorado hairstreak	-	Summer/Fall
Lycaenidae	Leptotes_marina	marine blue	-	Spring&Summer/Fall
Lycaenidae	Lycaeides_melissa	melissa blue	-	-
Lycaenidae	Lycaena_arota	tailed copper	-	Summer/Fall
Lycaenidae	Lycaena_heteronea	blue copper	-	Summer/Fall
Lycaenidae	Lycaena_rubidus	ruddy copper	-	-
Lycaenidae	Ministrymon_leda	leda ministreak	-	Summer/Fall
Lycaenidae	Plebejus_acmon_	acmon blue	-	Spring&Summer/Fall
Lycaenidae	Plebejus_icarioides	boisduval's blue	-	Summer/Fall
Lycaenidae	Plebejus_saepiolus	greenish blue	-	Summer/Fall
Lycaenidae	Satyrium_behrii	behr's hairstreak	-	Summer/Fall
Lycaenidae	Strymon_istapa	mallow scrub hairstreak	-	Summer/Fall
Lycaenidae	Strymon_melinus	gray hairstreak	-	Spring&Summer/Fall
Lycaenidae	Zizula_cyna	cyna blue	Summer/Fall	-
**Nymphalidae**	**Adelpha_bredowii***	**arizona sister**	**-**	**Summer/Fall**
Nymphalidae	Agraulis_vanillae	gulf fritillary	-	Spring&Summer/Fall
Nymphalidae	Anaea_aidea	tropical leafwing	-	Summer/Fall
Nymphalidae	Anaea_andria	goatweed leafwing	-	-
Nymphalidae	Anthanassa_campestris*	field crescent	-	Summer/Fall
Nymphalidae	Anthanassa_mylitta*	mylitta crescent	-	Spring&Summer/Fall
Nymphalidae	Anthanassa_picta*	painted crescent	-	Summer/Fall
Nymphalidae	Anthanassa_texana*	texan crescent	-	Spring&Summer/Fall
Nymphalidae	Anthanassa_tharos*	pearl crescent	-	Summer/Fall
Nymphalidae	Anthanassa_vesta*	vesta crescent	-	Summer/Fall
Nymphalidae	Asterocampa_celtis	hackberry emperor	-	Summer/Fall
Nymphalidae	Asterocampa_clyton	tawny emperor	-	Summer/Fall
Nymphalidae	Asterocampa_leilia	empress leilia	-	Spring&Summer/Fall
Nymphalidae	Cercyonis_meadii	mead's wood nymph	-	-
Nymphalidae	Cercyonis_oetus	small wood nymph	-	Summer/Fall
Nymphalidae	Cercyonis_sthenele	great basin wood nymph	-	Summer/Fall

Nymphalidae	Chlosyne_acastus	sagebrush checkerspot	-	Spring&-
Nymphalidae	Chlosyne_californica	california patch	-	Spring&Summer/Fall
Nymphalidae	Chlosyne_lacinia	bordered patch	Spring	Summer/Fall
Nymphalidae	Coenonympha_tullia	common ringlet	-	-
Nymphalidae	Cyllopsis_pertepida	canyonland satyr	-	Summer/Fall
Nymphalidae	Cyllopsis_pyracmon	nabokov's satyr	-	Summer/Fall
**Nymphalidae**	**Danaus_eresimus**	**soldier**	**Summer/Fall**	**-**
Nymphalidae	Danaus_gilippus	queen	-	Spring&Summer/Fall
Nymphalidae	Danaus_plexippus	monarch	-	Spring&Summer/Fall
Nymphalidae	Dymasia_dymas	tiny checkerspot	-	Spring&Summer/Fall
Nymphalidae	Euphydryas_chalcedona	variable checkerspot	-	Spring&Summer/Fall
Nymphalidae	Euptoieta_claudia	variegated fritillary	-	Spring&Summer/Fall
Nymphalidae	Euptoieta_hegesia	mexican fritillary	Summer/Fall	-
Nymphalidae	Gyrocheilus_patrobas	red bordered satyr	-	Summer/Fall
Nymphalidae	Junonia_coenia*	common buckeye	-	Spring&Summer/Fall
Nymphalidae	Libytheana_carinenta	american snout	-	Spring&Summer/Fall
Nymphalidae	Limenitis_archippus	viceroy	Spring	Summer/Fall
Nymphalidae	Limenitis_arthemis	red spotted admiral	-	-
Nymphalidae	Limenitis_weidemeyerii	weidemeyer's admiral	-	Summer/Fall
**Nymphalidae**	**Marpesia_petreus**	**ruddy daggerwing**	**-**	**-**
Nymphalidae	Megisto_rubricata	red satyr	-	Summer/Fall
Nymphalidae	Mestra_amymone	common mestra	Summer/Fall	-
Nymphalidae	Microtia_elva	elf	-	Summer/Fall
Nymphalidae	Myscelia_cyananthe	blackened bluewing	-	-
Nymphalidae	Nymphalis_antiopa	mourning cloak	-	Spring&Summer/Fall
Nymphalidae	Nymphalis_californica	california tortoiseshell	-	-
Nymphalidae	Nymphalis_milberti	milbert's tortoiseshell	-	-
Nymphalidae	Paramacera_allyni	pine satyr	-	Summer/Fall
Nymphalidae	Polydryas_arachne	arachne checkerspot	-	-

Nymphalidae	Polygonia_gracilis	zephyr hoary comma	-	-
Nymphalidae	Polygonia_interrogationis	question mark	-	Summer/Fall
Nymphalidae	Polygonia_satyrus	satyr comma	-	Spring&Summer/Fall
Nymphalidae	Speyeria_atlantis	atlantis fritillary	-	Summer/Fall
Nymphalidae	Texola_elada	elada checkerspot	-	Spring&Summer/Fall
Nymphalidae	Thessalia_cyneas	black checkerspot	-	Spring&Summer/Fall
Nymphalidae	Thessalia_fulvia	fulvia checkerspot	-	Spring&-
Nymphalidae	Thessalia_theona	theona checkerspot	-	Summer/Fall
Nymphalidae	Vanessa_annabella	west coast lady	-	Spring&Summer/Fall
Nymphalidae	Vanessa_atalanta	red admiral	-	Spring&Summer/Fall
Nymphalidae	Vanessa_cardui	painted lady	-	Summer/Fall
Nymphalidae	Vanessa_virginiensis	american lady	-	Summer/Fall
Papilionidae	Battus_philenor	pipevine swallowtail	-	Summer/Fall
Papilionidae	Papilio_indra	indra swallowtail	-	-
Papilionidae	Papilio_machaon*	baird's old world swallowtail	-	Summer/Fall
Papilionidae	Papilio_multicaudata	two tailed swallowtail	-	Summer/Fall
Papilionidae	Papilio_polyxenes	black swallowtail	-	Summer/Fall
Papilionidae	Papilio_rumiko	giant swallowtail	-	-
Papilionidae	Papilio_rutulus	western tiger swallowtail	-	Summer/Fall
Pieridae	Anteos_clorinde	white angled sulphur	Summer/Fall	-
Pieridae	Anthocharis_cethura	pima orangetip	-	-
Pieridae	Anthocharis_sara	sara orangetip	-	-
Pieridae	Appias_drusilla	florida white	-	-
Pieridae	Ascia_monuste	great southern white	-	Summer/Fall
Pieridae	Colias_cesonia	southern dogface	-	Summer/Fall
Pieridae	Colias_eurytheme	orange sulphur	-	Summer/Fall
Pieridae	Colias_philodice	clouded sulphur	-	Summer/Fall
Pieridae	Euchloe_hyantis	desert pearly marble	-	-
Pieridae	Eurema_boisduvaliana	boisduval's yellow	-	Summer/Fall
Pieridae	Eurema_daira	barred yellow	-	-
Pieridae	Eurema_dina	dina yellow	-	-
Pieridae	Eurema_lisa	little yellow	-	-
Pieridae	Eurema_mexicana	mexican yellow	-	Summer/Fall

Pieridae	Eurema_nicippe	sleepy orange	-	Summer/Fall
Pieridae	Eurema_nise	mimosa yellow	-	Summer/Fall
Pieridae	Eurema_proterpia	tailed orange	-	Summer/Fall
Pieridae	Kricogonia_lyside	lyside sulphur	-	Summer/Fall
Pieridae	Nathalis_iole	dainty sulphur	-	Summer/Fall
Pieridae	Neophasia_menapia	pine white	-	Summer/Fall
Pieridae	Neophasia_terlootii	chiricahua white	-	Summer/Fall
Pieridae	Phoebis_agarithe	large orange sulphur	-	Summer/Fall
Pieridae	Phoebis_philea	orange barred sulphur	-	-
Pieridae	Phoebis_sennae	cloudless sulphur	-	Summer/Fall
Pieridae	Pieris_napi	margined mustard white	Summer/ Fall	-
Pieridae	Pieris_rapae	cabbage white	-	Summer/Fall
Pieridae	Pontia_beckerii	becker's white	-	-
Pieridae	Pontia_protodice	checkered white	-	Summer/Fall
Pieridae	Pontia_sisymbrii	spring white	-	-
Riodinidae	Apodemia_hepburni	hepburn's metalmark	-	-
Riodinidae	Apodemia_mormo	mormon metalmark	-	Summer/Fall
Riodinidae	Apodemia_nais	nais metalmark	-	Summer/Fall
Riodinidae	Apodemia_palmeri	palmer's metalmark	-	Summer/Fall
Riodinidae	Calephelis_arizonensis	arizona metalmark	-	Summer/Fall
Riodinidae	Calephelis_nemesis	fatal metalmark	Spring	Summer/Fall
Riodinidae	Emesis_ares	ares metalmark	-	Summer/Fall
Riodinidae	Emesis_zela	zela metalmark	-	Summer/Fall

*Note the following name changes from how they were identified in the NABA dataset to listings in this table and considered in the dataset (NABA to corrected name): *Adelpha eulalia* to *Adelpha bredowii*, *Junonia genoveva* to *Junonia coenia*, *Papilio machaon bairdii* to Papilio machaon, *Papilio cresphontes* to *Papilio Rumiko, genus Phyciodes to Anthanassa*

Table S3. Climate variables and descriptions considered for inclusion in the final model. These variables were all included in the forward stepwise regression to identify the final predictor variables in the final model

Variable(s)	Description
Maximum Temperature	The highest recorded temperature (°C) for the survey day
Maximum Temperature of the previous 30/90/365 Days	The warmest temperature (°C) recorded in the 30/90/365 days prior to the survey date
Mean Temperature	The mean temperature (°C) for the survey day

Mean Temperature of the Previous 30/90/365 Days	The mean temperature (°C) over the 30/90/365 days prior to the survey date
Minimum Temperature	The lowest recorded temperature (°C) for the survey day
Minimum Temperature of the Previous 30/90/365 Days	The coldest temperature (°C) recorded in the 30/90/365 days prior to the survey date
Monsoon Season Maximum Temperature	The maximum temperature (°C) in the monsoon season (July-September) prior to the survey date
Monsoon Season Mean Temperature	The mean temperature (°C) in the monsoon season (July-September) prior to the survey date
Monsoon Season Minimum Temperature	The minimum temperature (°C) in the monsoon season (July-September) prior to the survey date
Monsoon Season Precipitation	The total precipitation (mm) in the monsoon season (July-September) prior to the survey date
Precipitation	Precipitation on the survey day (mm)
Precipitation total of the previous 30/90/365 days	The precipitation total (mm) in the 30/90/365 days prior to the survey date
Previous 30/90/365 Days above 28	The total number of days that reached a temperature above 28 (°C) in the 30/90/365 days prior to the survey date
Previous 30/90/365 Days above 30	The total number of days that reached a temperature above 30 (°C) in the 30/90/365 days prior to the survey date
Recent Precipitation 1	The precipitation (mm) of the most recent rainy season relative to the sampling date.For March to July, the most recent was the winter rains, for August and September, the most recent was the previous 90 days, and for October, the most recent was the recent monsoon rains
Recent Precipitation 2	The same as Recent Precipitation 1, except the most recent rains for August were the previous winter rains.
Winter Season Maximum Temperature	The maximum temperature (°C) in the winter season (October-April) previous to the survey date
Winter Season Mean Temperature	The mean temperature (°C) in the winter season (October-April) previous to the survey date

Winter Season Minimum Temperature	The minimum temperature (°C) in the winter season (October-April) previous to the survey date
Winter Season Precipitation	The total precipitation (mm) in the Winter season (October-April) previous to the survey date

Table S4. Linear mixed model output table for summer/fall survey butterfly abundance. Site was included as a random effect in this model. Asterisks represent significance at the following levels: *** = p < 0.001; ** = p < 0.01; * = p < 0.05; ^ = p < 0.10.

Fixed Effects	Estimate	Standard Error	Degrees of Freedom	t Value	Pr(>|t|)
Intercept	41.7007	13.220	148.634	3.154	0.0019 **
Year	-0.0181	0.0066	143.789	-2.725	0.0072**
Min Temp (Previous 30 days)	0.0342	0.0416	79.982	0.821	0.4140
Max Temp (Previous 30 days)	-0.0283	0.0364	168.141	-0.777	0.4384
Monsoon Precipitation	0.0024	0.0007	173.693	3.438	0.0007 **
Winter Precipitation	-0.0001	0.0005	172.885	-0.097	0.9227
Party Hours	0.0360	0.0044	81.775	8.074	<0.0001 ***

Significance codes: 0 '***' 0.001 '**' 0.01 '*' 0.05 '.'

Table S5. General linear model output table for spring survey butterfly abundance. Asterisks represent significance at the following levels: *** = p < 0.001; ** = p < 0.01; * = p < 0.05; ^ = p < 0.10.

Fixed Effects	Estimate	Standard Error	t Value	Pr(>|t|)
Intercept	197.4916	105.355	1.875	0.0903 ^
Year	-0.0949	0.0517	-1.836	0.0962 ^
Min Temp (Previous 30 days)	-0.0294	0.0539	-0.545	0.5978
Max Temp (Previous 30 days)	-0.0826	0.0786	-1.050	0.3184
Monsoon Precipitation	-0.0002	0.0013	-0.152	0.8821
Winter Precipitation	0.0037	0.0014	2.703	0.0221 *
Party Hours	0.0472	0.0098	4.821	0.0007 ***

Table S6. General linear model output table for spring survey butterfly richness. Asterisks represent significance at the following levels: *** = p < 0.001; ** = p < 0.01; * = p < 0.05; ^ = p < 0.10.

Fixed Effects	Estimate	Standard Error	t Value	Pr(>|t|)
Intercept	4331.1170	1146.1705	3.779	0.0036 **
Year	-2.1393	0.5625	-3.803	0.0035 **
Min Temp (Previous 30 days)	-0.8376	0.5874	-1.426	0.1843
Max Temp (Previous 30 days)	-0.0968	0.8561	-0.113	0.9122
Monsoon Precipitation	-0.0313	0.0144	-2.162	0.0560 ^
Winter Precipitation	0.0423	0.0151	2.784	0.0193 *
Party Hours	0.7466	0.1065	7.007	<0.0001 ***

Table S7. Linear mixed model output table for summer/fall survey butterfly richness. Site was included as a random effect in this model. Asterisks represent significance at the following levels: *** = p < 0.001; ** = p < 0.01; * = p < 0.05; ^ = p < 0.10.

Fixed Effects	Estimate	Standard Error	Degrees of Freedom	t Value	Pr(>|t|)
Intercept	-2.636	13.5925	173.1268	-0.194	0.8465
Year	0.0050	0.0068	1743.823	0.728	0.4676
Min Temp (Previous 30 days)	0.0148	0.0479	163.606	0.309	0.7574
Max Temp (Previous 30 days)	-0.0739	0.0367	172.3101	-2.011	0.0459 *
Monsoon Precipitation	0.0006	0.0007	169.3826	0.903	0.3680
Winter Precipitation	0.0016	0.0004	165.2866	3.294	0.0012 ***
Party Hours	0.0390	0.0049	174.9570	7.956	<0.0001 ***

Table S8. Variance Inflation Factor (VIF) Output for each model. Summer/fall models have different VIF values for the abundance and richness models because they include a random factor.

Model	Year	Min Temp (Previous 30 days)	Max Temp (Previous 30 days)	Monsoon Precipitation	Winter Precipitation	Party Hours
Summer/Fall Abundance	1.097	1.189	1.399	1.203	1.109	1.030
Fall Richness	1.157	1.112	1.234	1.173	1.117	1.044
Spring Abundance and Richness	1.441	1.930	1.963	1.823	1.247	2.364
